# Quantifying trade-offs: quality of life and quality-adjusted survival in a randomised trial of chemotherapy in postmenopausal patients with lymph node-negative breast cancer

**DOI:** 10.1038/sj.bjc.6602230

**Published:** 2004-11-16

**Authors:** J Bernhard, D Zahrieh, A S Coates, R D Gelber, M Castiglione-Gertsch, E Murray, J F Forbes, L Perey, J Collins, R Snyder, C-M Rudenstam, D Crivellari, A Veronesi, B Thürlimann, M F Fey, K N Price, A Goldhirsch, C Hürny

**Affiliations:** 1 IBCSG Coordinating Center, Bern, Switzerland; 2 Institute of Medical Oncology, Inselspital, Bern, Switzerland; 3 IBCSG Statistical Center, Dana-Farber Cancer Institute, Boston, MA, USA; 4 Frontier Science and Technology Research Foundation, Boston, MA, USA; 5 The Cancer Council Australia, Australia; 6 University of Sydney, Australia; 7 Groote Schuur Hospital, South Africa; 8 University of Cape Town, South Africa; 9 Australian New Zealand Breast Cancer Trials Group, Newcastle, Australia; 10 Newcastle Mater Misericordiae Hospital, Newcastle, Australia; 11 Centre Hospitalier Universitaire Vaudois, Lausanne; 12 Department of Surgery, The Royal Melbourne Hospital, Melbourne, Australia; 13 Department of Oncology, St Vincent's Hospital, Melbourne, Australia; 14 West Swedish Breast Cancer Study Group, Sahlgrenska University Hospital, Göteborg, Sweden; 15 Centro di Riferimento Oncologico, Aviano, Italy; 16 Kantonsspital, St Gallen, Switzerland; 17 Oncology Institute of Southern Switzerland, Lugano, Switzerland; 18 European Institute of Oncology, Milan, Italy; 19 Bürgerspital, St Gallen, Switzerland

**Keywords:** breast cancer, adjuvant therapy, quality of life, quality-adjusted survival, Q-TWiST, utility

## Abstract

We evaluated quality of life (QL) and quality-adjusted survival in International Breast Cancer Study Group Trial IX, a randomised trial including 1669 eligible patients receiving tamoxifen for 5 years or three prior cycles of cyclophosphamide, methotrexate and 5-fluorouracil (CMF) followed by 57 months tamoxifen. During the time with CMF toxicity (Tox), without symptoms and toxicity (TWiST), and following relapse (Rel), patients scored their QL indicators and a utility indicator for subjective health estimation between ‘perfect’ and ‘worst’ health. Scores were averaged within Tox, TWiST and Rel and transformed to utilities. Mean durations for the three transition times were weighted with utilities to obtain mean quality-adjusted TWiST (Q-TWiST). Patients receiving CMF reported significantly worse scores for most QL domains at month 3, but less hot flushes. After completing chemotherapy, there were no differences by treatment groups. Benefits evaluated by Q-TWiST favoured the additional chemotherapy. CMF provided 3 more months of Q-TWiST for patients with ER-negative tumours, but CMF provided no benefit in Q-TWiST for patients with ER-positive tumours. Q-TWiST analysis based on patient ratings is feasible in large-scale cross-cultural clinical trials.

The International Breast Cancer Study Group (IBCSG) recently presented first results of a randomised trial (Trial IX) testing the role of adjuvant chemotherapy preceding treatment with tamoxifen for postmenopausal patients with lymph node-negative breast cancer (International Breast Cancer Study Group, 2002). Patients showed substantially better disease-free survival (DFS) with adjuvant chemotherapy if their oestrogen receptor (ER) status was negative (i.e. endocrine nonresponsive). In contrast, if their cancer was ER-positive (i.e. endocrine-responsive), they obtained no benefit from the chemotherapy compared with tamoxifen alone. In this report, we extend the analysis to quantify trade-offs based on quality of life (QL) and quality-adjusted survival (QAS).

The objective of the QL analysis was an extension of our earlier findings on the impact of adjuvant therapy on QL (Hürny *et al*, 1996b). Presence, duration, timing and anticipation of chemotherapy had a measurable effect on patients' QL, but patients' psychological adaptation was found to be more important for their QL than cytotoxic side effects. In this trial, we used additional QL indicators (Bernhard *et al*, 1997).

The objective of the QAS analysis was an extension of the Q-TWiST model designed to evaluate ‘trade-offs’ in clinical trials (Goldhirsch *et al*, 1989). This model divides the life-span from the beginning of adjuvant treatment until death into three time segments corresponding to distinct health states: Tox (time with toxicity), TWiST (time without symptoms and toxicity) and Rel (time after systemic relapse). In previous trials, Tox and Rel were weighted by arbitrary utilities and added to TWiST to reach an overall assessment of different treatment groups. In this trial, patients were asked to directly assess the relevant utilities during Tox, TWiST and Rel using a scale which asked them to imagine that they would have to live the rest of their life in their current condition and then to rate this condition between ‘perfect health’ and ‘worst health’. This scale has been shown to correspond to utility values as measured by conventional time trade-off (Hürny *et al*, 1998).

QL and QAS are usually evaluated separately despite their complementary objectives: understanding the pattern of morbidity and adaptation (QL), and providing information for clinical policy and decision-making (QAS). In this analysis, we link these two concepts to provide a quantitative backing for the trade-offs inherent in the use of effective but toxic therapies.

## PATIENTS AND METHODS

### The Trial

Between October 1988 and August 1999, 1715 postmenopausal patients were randomly assigned to receive either tamoxifen (20 mg day^−1^) for 60 months or three 28-day courses of CMF (cyclophosphamide at 100 mg m^−2^ orally on days 1–14, methotrexate at 40 mg m^−2^ intravenously on days 1 and 8 and 5-fluorouracil at 600 mg m^−2^ intravenously on days 1 and 8, followed by tamoxifen (20 mg day^−1^) for 57 months. Tamoxifen following chemotherapy was to begin on day 15 of the final course of CMF. Of the 1715 patients randomly assigned, 1669 (97%) were eligible and assessable. The details of the trial protocol and conduct are described elsewhere (International Breast Cancer Study Group, 2002). The median follow-up of this analysis was 71 months. Participating investigators are displayed in Appendix A.

Patients were asked to complete a QL form at beginning of treatment (baseline), 2 months later (i.e. day 1 of cycle 3 or 8 weeks after tamoxifen start), at each 3 months for the first year, at months 18 and 24, and 1 and 6 months after relapse. This schedule was expanded, with yearly assessments up to month 72 (June 1995) regardless of the disease status (December 1996), that is, the 1 and 6 month assessments following relapse were dropped.

### Quality of life analysis

We predicted worse QL during chemotherapy (month 3) but no residual effects among treatment groups after completion of chemotherapy (Hürny *et al*, 1996b). A clinically meaningful difference was defined based on our previous observations of the coping indicator in postmenopausal patients with lymph node-positive breast cancer (Hürny *et al*, 1996b), as the ratio of the average change between baseline and month 3 in patients with Tam alone *vs* those with three initial cycles CMF followed by Tam (ratio=3.37; not shown in the original article). We chose to use the ratio instead of the absolute difference in scores because the baseline scores in the present trial were better than those of the earlier trial, which involved women with node-positive disease.

As a secondary hypothesis, we expected smaller adverse effect of CMF on QL at month 3 for patients with ER-negative than those with ER-positive tumours. We reasoned that patients with ER-negative tumours would be more likely to feel that chemotherapy was necessary and therefore find it more bearable than patients with ER-positive disease.

We report QL data for the first 24 months in patients without recurrence within this time. Of the 1669 eligible and assessable patients, 1398 patients were included in the QL analysis. Of these, 803 completed all of their assessments using the 1993 version of the IBCSG QL Core Form (Bernhard *et al*, 1997). This form comprised indicators for physical well being, mood (Hürny *et al*, 1996a), coping effort (Hürny *et al*, 1993), appetite, tiredness, hot flushes, nausea/vomiting, perceived social support, restrictions in arm movement and subjective health estimation (SHE) (Hürny *et al*, 1998) in the linear analogue self-assessment (LASA) format. The other 595 patients either filled out all of their assessments using the previous version of the form, which included four of these indicators (physical well being, mood, coping effort, appetite) (Butow *et al*, 1991), or they filled out a combination of the two. For consistency, only data provided by the 803 patients mentioned above were used for the analyses of the additional indicators. All indicators were analysed separately.

To test for differences between treatments in QL scores at each time point, we used ANOVA adjusting for culture (country/language; see Table 1

**Table 1:** Description of patients excluded from the quality of life (QL) analysis

Total eligible cases	1669
	
*Exclusions from analysis*	
Relapse within first 24 months of randomisation	136
Completed no QL forms	98
Completed QL forms in multiple languages	33
Undefined culture^a^	4
	
Assessable cases	1398^b^

a Culture is defined by the following language/country combinations: English/Australia; New Zealand; English/South Africa; French/Switzerland; German/Switzerland; Italian/Switzerland; Italian/Italy; Slovenian/Slovenia; Spanish/Spain; Swedish/Sweden.

b Of the 1398 patients, 803 completed all of their assessments using the 1993 version of the QL form. These 803 patients were used for the analyses of indicators included alone on this version of the form (tiredness, hot flushes, nausea/vomiting, social support, arm movement and SHE). All 1398 patients were used for the analyses of indicators included on both versions of the QL form (physical well being, mood, appetite and PACIS).

for definitions). For baseline analysis and tests for heterogeneity among treatment groups at each time point, we used the square roots of the scores (Hürny *et al*, 1996b) because this transformation approximated a normal distribution and was effective in stabilising the variances for all indicators. The figures however show the results in the original scores from 0 to 100.

We also tested for differences in QL scores between baseline and month 3 and 6, respectively, of the within-patient changes in an ANOVA model that included assigned treatment and culture. The intrapatient differences were normally distributed for all QL scores.

### Quality-adjusted survival analysis

For this analysis, we used the SHE indicator as designed for QAS (Hürny *et al*, 1998). Patients were asked to imagine that they would have to live the rest of their life in their current condition and then to rate this condition between ‘perfect health’ and ‘worst health’. This indicator was previously validated against a time trade-off (TTO) interview (i.e. a preference measure) in patients with metastatic breast cancer. The conventional negative anchor ‘death’ was replaced by ‘worst health’ in the adjuvant setting since the two versions showed comparable results and ‘death’ was judged to be an unacceptable anchor for some of the language/culture groups in the present study.

Following the Q-TWiST model (Goldhirsch *et al*, 1989), we defined three clinical health states: Tox, TWiST and Rel. The clinical question of this trial was the impact of adding three cycles of CMF. Therefore, Tox was calculated only in patients randomised to receive CMF.

To calculate Q-TWiST, each health state is assigned a utility coefficient (*u*_t_, *u*_twist_ and *u*_rel_) which gives a value to time spent in the state relative to the value of an equal amount of time spent in a state of ‘perfect health’. The utilities are assumed to be in the interval [0,1], where a zero indicates worst possible health, and unity indicates a state as good as perfect health. The Q-TWiST model is obtained by taking the linear combination of the health state durations adjusted by the respective utilities:


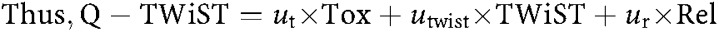


Our objective was to make a realistic patient-derived estimate of the utilities to evaluate Q-TWiST. We hypothesised that utilities derived from observed SHE values in both the Tox and Rel states would be substantially higher than the arbitrary value of 0.5 used to illustrate the Q-TWiST method when it was introduced (Goldhirsch *et al*, 1989). Also, we predicted that the actual scores during the TWiST state would be less than ‘perfect’, if only because all patients were receiving Tam.

Within each defined health state, all available SHE scores were used (*N*=1669). For Tox, we used the median value at month 3 (i.e. peak of toxicity). For TWiST, we used the median of the SHE scores averaged within patients over the first 24 months after randomisation (excluding the first 3 months in patients with CMF). For Rel, we used the median of the SHE scores averaged within patients over the first 6 months after relapse. These SHE estimates were converted to quality weights using a power transformation: TTO=1–(1–SHE)^*α*^ (Torrance *et al*, 1996; Torrance *et al*, 2001). We used the *α* value from our validation study (*α*=1.6) and performed a sensitivity analysis for a range of published *α*'s.

An exploratory analysis was conducted to assess *u*_t_ confined to those patients who had any recorded subjective toxic effect of grade 2 or higher (excluding amenorrhea) during their chemotherapy as was done in the original Q-TWiST model (Goldhirsch *et al*, 1989).

Mean health state durations were estimated from censored survival data (product limit method) up to 72 months from randomisation by computing the areas between the survival curve estimates for the transition times. These durations were adjusted using the patient-derived utilities in order to estimate mean Q-TWiST for each treatment group. The treatments were separately compared overall and within the prospectively stratified ER-negative and ER-positive cohorts.

We performed a threshold utility analysis both overall and within the ER-negative and ER-positive cohorts. These findings provide a decision aid for a range of utilities for Tox and Rel. To account for the less than ‘perfect health’, we divided each of the three utilities by the patient estimated *u*_twist_ so that Q-TWiST is interpreted relative to TWiST and more accurately reflects the patients’ perception during the time period: Q-TWiST=*u*_t_/*u*_twist_ × Tox+*u*_twist_/*u*_twist_ × TWiST × *u*_r_/*u*_twist_ × Rel. The treatment comparison results are presented for all possible values of the utilities.

For all analyses, *P*-values less than 0.05 were deemed statistically significant. No adjustment was made for multiple testing.

## RESULTS

### Quality of life

Of the 1669 eligible patients, 1398 were assessable for the QL study. The submission rate of the QL form was 87% (1213) at baseline declining to 75% (1044) at month 24. The definition of the sample is shown in Table 1. The patient characteristics are shown in Table 2

**Table 2:** Baseline characteristics of the 1398 patients with available QL data

	**Tam**	**CMF → Tam**	**Total**
Total eligible cases	701 (100)	697 (100)	1398 (100)
ER-negative	147 (21)	149 (21)	296 (21)
ER-positive	525 (75)	516 (74)	1041 (74)
ER-unknown	29 (4)	32 (5)	61 (4)
			
Total mastectomy	332 (47)	350 (50)	682 (49)
Breast conservation	369 (53)	347 (50)	716 (51)
With planned RT	322 (87)	313 (90)	635 (89)
Without planned RT	47 (13)	34 (10)	81 (11)
			
Tumour size⩽1.0 cm	78 (11)	84 (12)	162 (12)
Tumour size 1.1–2.0 cm	336 (48)	335 (48)	671 (48)
Tumour size>2.0 cm	262 (37)	252 (36)	514 (37)
Tumour size unknown	25 (4)	26 (4)	51 (4)
			
Tumour grade 1	131 (19)	123 (18)	254 (18)
Tumour grade 2	294 (42)	285 (41)	579 (41)
Tumour grade 3	234 (33)	242 (35)	476 (34)
Tumour grade unknown	42 (6)	47 (7)	89 (6)
			
Vessel invasion absent	540 (77)	528 (76)	1068 (76)
Vessel invasion present	147 (21)	151 (22)	298 (21)
Vessel invasion not examined	14 (2)	18 (2)	32 (2)
			
Age (median [range])	61 [34–78]	60 [44–81]	61 [34–81]

.

Figure 1

**Figure 1:**
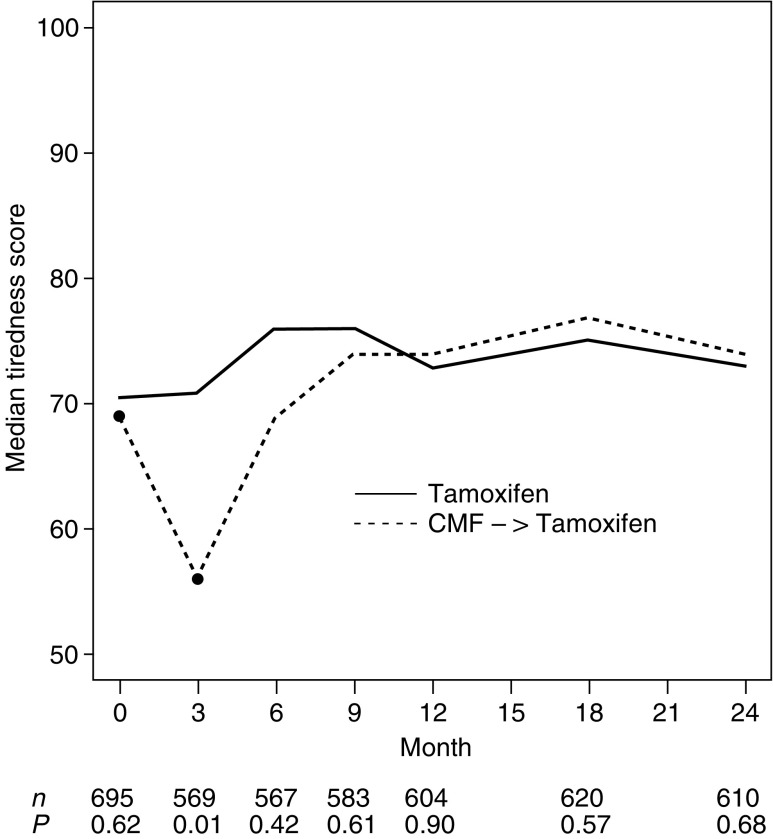
Median tiredness scores by treatment group over the first 24 months. Higher values indicate less tiredness (i.e. better QL).

depicts the expected short-term effect between treatments at month 3, with less severe tiredness (*P*=0.01) in patients receiving Tam alone, as compared to those patients with three initial cycles of CMF. Nausea/vomiting (*P*<0.01) and appetite were similarly affected (*P*<0.01). Also, at 3 months, physical well being (*P*<0.01) and mood (*P*<0.01) showed effects in favour of Tam alone. Subjective health estimates (SHE) were similarly affected (*P*=0.02), as displayed in Figure 2

**Figure 2:**
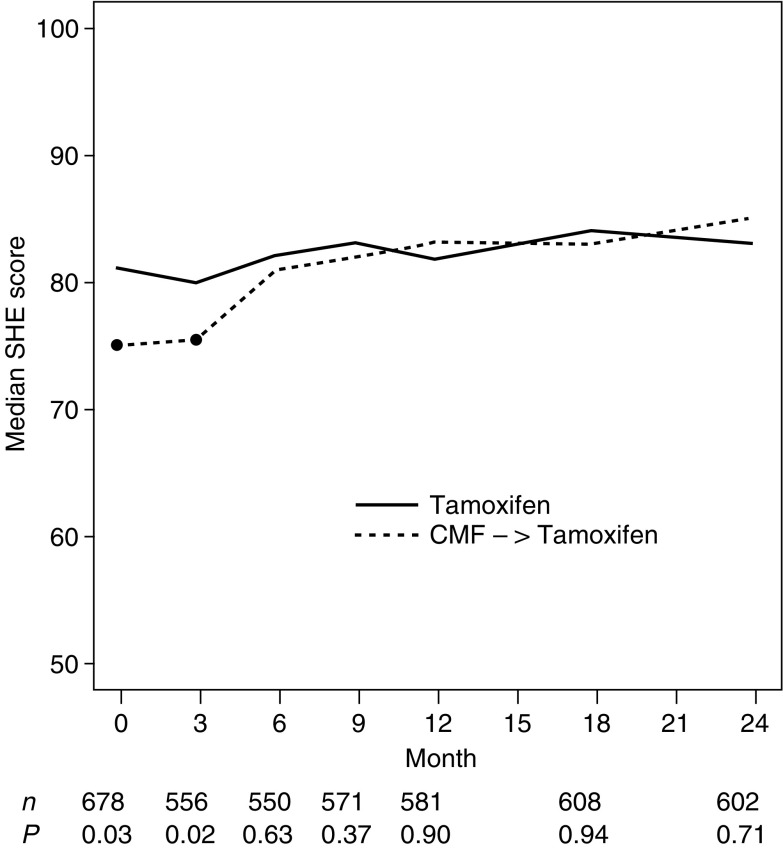
Median scores of subjective health (SHE) by treatment group over the first 24 months. Higher values indicate better health estimates.

. In contrast, patients receiving CMF reported less hot flushes at 3 months (*P*<0.01) than those receiving Tam. CMF recipients started tamoxifen after completing chemotherapy.

**Figure 3:**
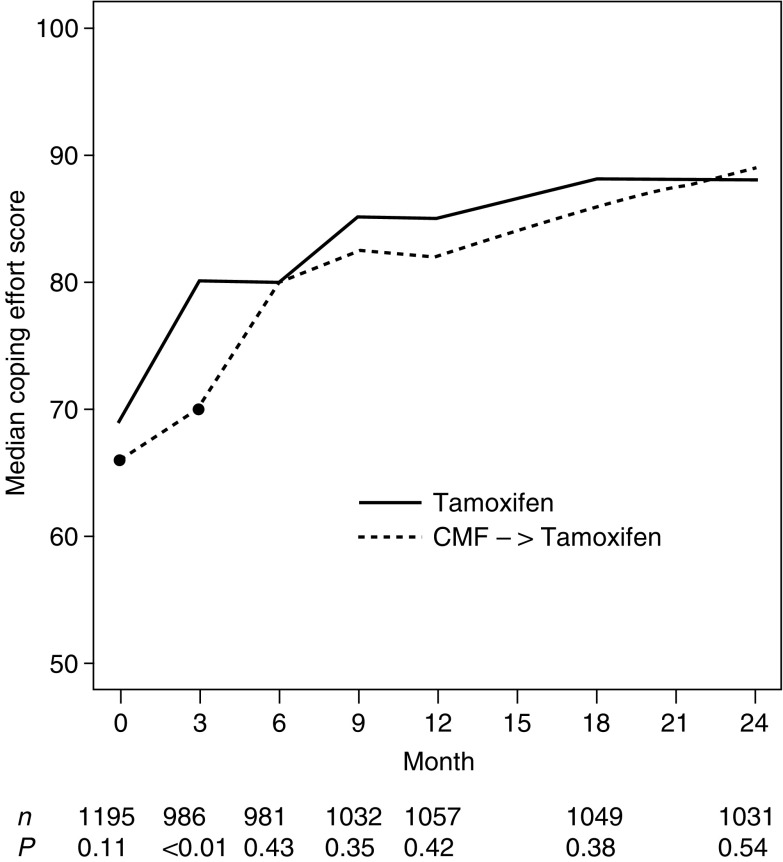
Median scores of coping effort (PACIS) by treatment group over the first 24 months. Higher values indicate less effort to cope (i.e. better QL).

The global QL indicators showed effects in favour of Tam alone. The coping scores indicated a steady improvement over the first 24 months. This adaptation was delayed but not prevented in patients receiving CMF (*P*<0.01), as shown in Figure 3

. The ratio of the average change in the coping indicator between baseline and month 3 in patients with Tam alone *vs* those with CMF followed by Tam (ratio=3.17) was close to that observed in our previous trial (ratio=3.37), indicating a clinically meaningful difference. There was a baseline difference (*P*=0.03) with patients assigned to CMF reporting lower SHE scores. Of these patients, 43% completed the QL form prior to randomisation (thus up to 57% did so after knowing treatment assignment), and 95% of all patients with CMF at day 1 of the first cycle or before (thus 5% did so after experiencing initial toxicity).

**Figure 4:**
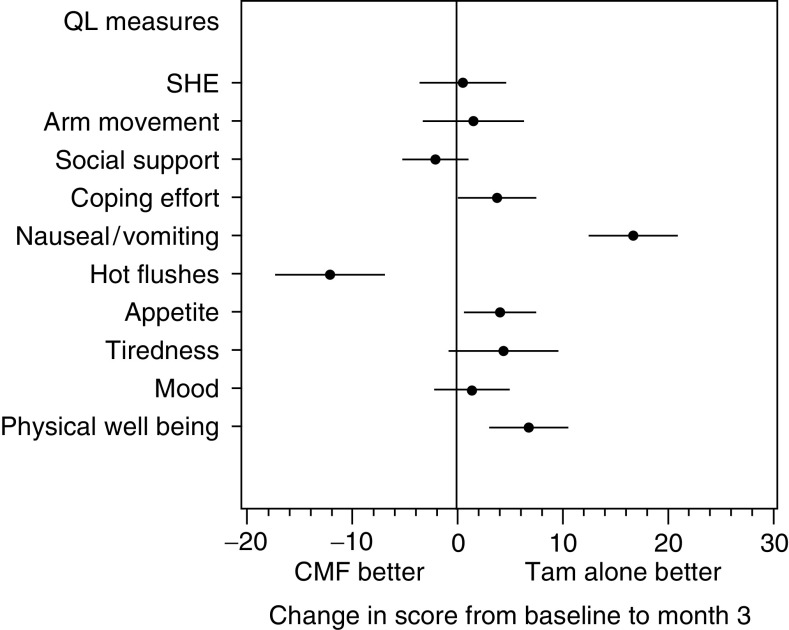
Mean treatment differences over the first 3 months with their respective 95% confidence intervals (*N*=914). Positive values indicate either an improvement or less deterioration in QL for patients randomised to receive Tam alone compared with patients randomised to receive CMF followed by Tam.

The mean treatment differences in the changes of scores over the first 3 months are shown in Figure 4

. After the completion of chemotherapy (i.e. month 6 assessment), there were no statistically significant residual differences between treatment groups for any of the QL measures.

Similar analyses were performed within the ER-negative and ER-positive cohorts. Contrary to our hypothesis, there was no indication for a different treatment effect on QL by ER status (data not shown).

## QUALITY-ADJUSTED SURVIVAL

Patients assigned to CMF had improved disease-free survival (DFS) compared with patients who received Tam alone ([CMF+Tam/Tam] RR=0. 80; 95% CI=0.64 to 1.00; *P*=0.05). There was also a trend in favour of improved overall survival (OS) ([CMF+Tam/Tam] RR=0.75; 95% CI=0.55 to 1.02; *P*=0.07). Disease-free survival and OS at 72 months for all 1669 eligible patients are summarised in Table 3

**Table 3:** Overall survival (OS)and DFS by treatment at 72 months (*N*=1669)

	**No. of patients**	**No. of events**	**6-year DFS% (s.e.)**	***P***	**No. dead**	**6-year OS% (s.e.)**	***P***
*Overall*							
Tam	846	166	78 (2)	0.05	94	87 (1)	0.07
CMF → Tam	823	138	83 (1)		73	91 (1)	
							
*ER-negative*							
Tam	190	57	67 (4)	0.003	38	75 (4)	0.01
CMF → Tam	192	33	83 (3)		21	88 (3)	
							
*ER-positive*							
Tam	621	101	81 (2)	0.92	51	90 (2)	0.80
CMF → Tam	596	101	82 (2)		50	92 (1)	

DFS=disease-free survival; s.e.=standard error.

.

The patient derived utilities for the three health states estimated from SHE scores are shown in Table 4

**Table 4:** Calculated utility coefficients

**Health state**	***N*^a^**	**SHE**	**TTO=1−(1−SHE)^1.6^**
			
*Tox (CMF)*			
Overall	276	0.76	0.89
ER-negative	59	0.75	0.89
ER-positive	217	0.76	0.90
			
*TWiST*			
Tam alone	384	0.80	0.91
CMF	358	0.80	0.91
ER-negative overall	161	0.80	0.91
ER-positive overall	578	0.80	0.91
			
*Rel*			
Tam alone	23	0.62	0.86
CMF	14	0.50	0.66
Total sample	37	0.54	0.71
ER-negative overall	12	0.54	0.71
ER-positive overall	24	0.56	0.73

a Sample size reflects those patients who experienced that health state and who responded to the SHE question at least once during that health state. SHE=subjective health estimation; TTO=time trade-off ; ER=oestrogen receptor.

. For Tox, 276 patients with CMF responded to the SHE question. The patient-derived utility for this period was 0.89. Of these patients, 66% (*N*=183) had a subjective toxic effect of grade 2 or higher (excluding amenorrhea) reported during the three cycles. The estimated utility for Tox of this subgroup was also 0.89.

For TWiST, SHE scores were available for 742 patients. The estimated utility for this period was 0.91. There was no difference in utility during TWiST by randomised treatment. For Rel, SHE scores were available for 37 patients. Those who relapsed after adjuvant CMF indicated a trend to lower SHE scores (median=0.5, range: 0.10–0.99) than those initially treated with Tam alone (median=0.62, range: 0.26–1.0). Given the small subgroups, we used the overall SHE scores (median=0.54) for QAS (*u*_r_=0.71).

The average values of time spent in Tox, TWiST and Rel within 72 months of randomisation are summarised in Table 5

**Table 5:** Components of Q-TWiST

	**Treatment group**				
**Health state**	**Tam alone (s.e.)**	**CMF → Tam (s.e.)**	**Difference (s.e.)**	**(95% CI)**	***P***
*Tox*					
Overall	0 (0)	1.7 (0.1)	—	—	—
ER-negative	0 (0)	1.8 (0.1)	—	—	—
ER-positive	0 (0)	1.7 (0.1)	—	—	—
					
*TWiST*					
Overall	63.4 (0.7)	64.0 (0.6)	—	—	—
ER-negative	57.7 (1.7)	62.6 (1.4)	—	—	—
ER-positive	65.4 (0.7)	64.2 (0.7)	—	—	—
					
*Rel*					
Overall	5.1 (0.5)	3.8 (0.4)	—	—	—
ER-negative	7.5 (1.2)	3.3 (0.9)	—	—	—
ER-positive	4.5 (0.6)	4.2 (0.6)	—	—	—
					
*Q-TWiST*					
Overall	61.4 (0.4)	62.5 (0.3)	1.1 (0.5)	(0.1–2.1)	0.03
ER-negative	57.8 (1.1)	60.8 (1.0)	3.0 (1.4)	(0.2–5.8)	0.03
ER-positive	62.7 (0.4)	63.0 (0.4)	0.3 (0.5)	(−0.6–1.3)	0.50

Averaged months of Tox, TWiST and Rel accumulated within 72 months of randomisation, with Q-TWiST calculated for patient-derived utility coefficients (*N*=1669). s.e.=standard error; ER=oestrogen receptor.

. The calculation of Q-TWiST is illustrated by the weighted combination of these components using the patient-derived estimates of *u*_t_=0.89, *u*_twist_=0.91 and *u*_r_=0.71. Based on these utilities, the average Q-TWiST within the first 6 years for patients receiving CMF followed by Tam was 62.5 months, 1.1 month longer than patients receiving Tam alone (*P*=0.03).

Patients benefited from adjuvant chemotherapy if their ER-status was negative, with an average gain in Q-TWiST of 3 months as compared to those with tamoxifen alone (*P*=0.03). Patients with ER-positive tumours obtained no Q-TWiST benefit from the chemotherapy .

Figure 5

**Figure 5:**
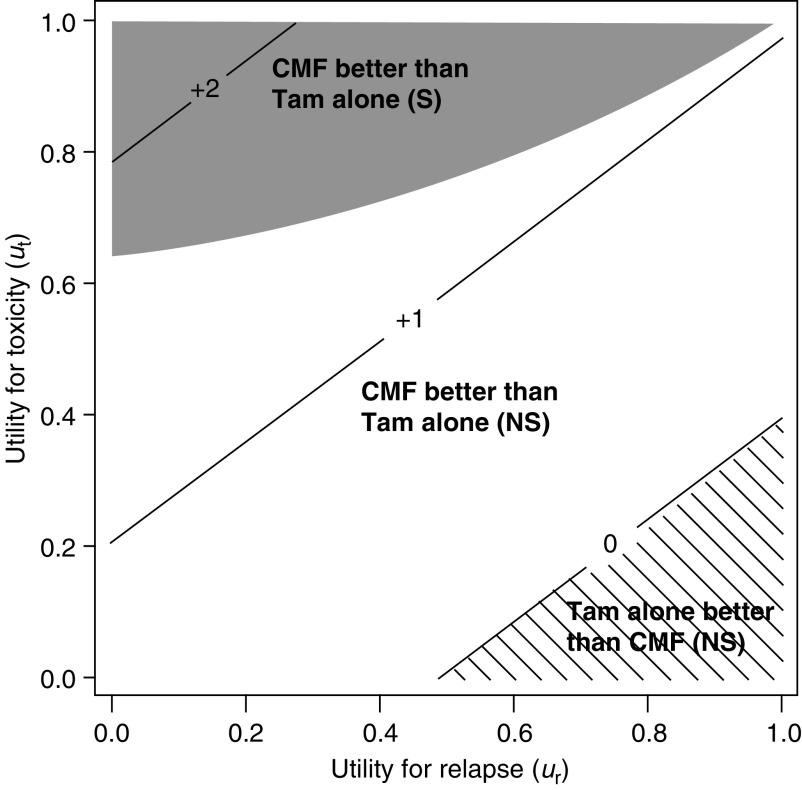
Threshold utility analysis of the total sample (*N*=1669). The diagonal lines indicate the units of months gained in Q-TWiST for different utilities for Tox and Rel. The utility for TWiST is defined as *u*_twist_=1 (i.e. reference state). The solid line labelled 0 is the threshold above which CMF followed by Tam results in improved Q-TWiST. The area below 0 with hatch marks represents improved Q-TWiST for Tam alone. The shaded region (top left) represents utilities (*u*_t_ and *u*_r_) for which the improvement in Q-TWiST is statistically significant (*P*⩽0.05). S=significant and NS=not significant.

allows a sensitivity analysis to display the effect on Q-TWiST of any combination of utilities during Tox and Rel. To prepare this figure, we standardised the other utilities relative to *u*_twist_ For example, a conventional treatment comparison for DFS makes no deduction for reduced utility during Tox (*u*_t_=*u*_twist_=1). Based on these assumptions, CMF followed by Tam is obviously preferred to Tam alone. Taking into account different values for Tox (*u*_t_<1;*u*_twist_=1) and including Rel, there is a gain in Q-TWiST for CMF followed by Tam for most values of *u*_t_ and *u*_r_. This trend towards an improvement reaches statistical significance for relatively high values of *u*_t_ as well as over a wide range of plausible values of *u*_r_ as displayed in the upper left portion of the figure. For low values of *u*_t_ combined with high values of *u*_r_, there is a nonsignificant benefit for tamoxifen alone as displayed in the lower right portion of the figure.

Figures 6

**Figure 6:**
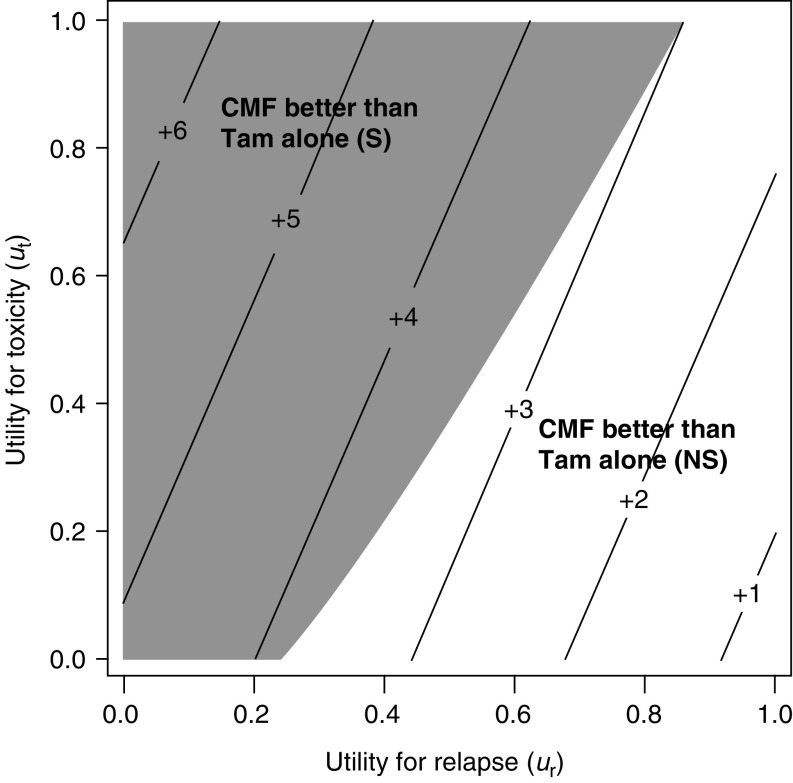
Threshold utility analysis of the ER-negative cohort (*N*=382). The diagonal lines indicate the units of months gained in Q-TWiST for different utilities for Tox and Rel. The utilities for TWiST is defined as *u*_twist_=1 (i.e. reference state). All values of the utilities for Tox and Rel result in improved Q-TWiST for CMF followed by Tam compared with Tam alone. The shaded region (top left) represents utilities (*u*_t_ and *u*_r_) for which the improvement in Q-TWiST is statistically significant (*P*⩽0.05). S=significant and NS=not significant.

and 7

**Figure 7:**
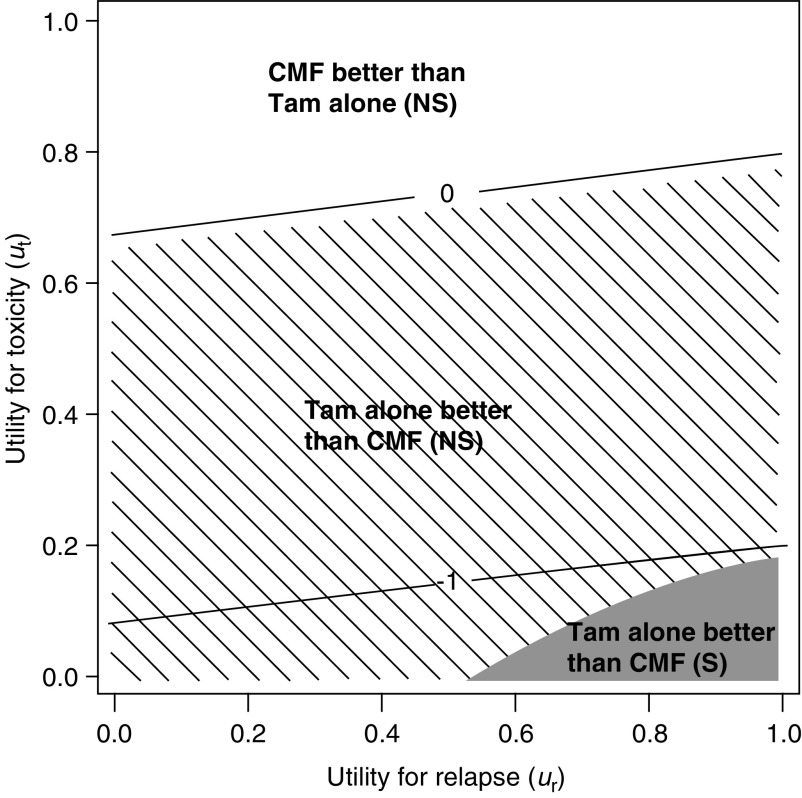
Threshold utility analysis of the ER-positive cohort (*N*=1217). The diagonal lines indicate the units of months gained in Q-TWiST for different utilities for Tox and Rel. The utilities for TWiST is defined as *u*_twist_=1 (i.e. reference state). The solid line labelled 0 is the threshold above which CMF followed by Tam results in improved Q-TWiST. The area below 0 with hatch marks represents improved Q-TWiST for Tam alone. The shaded region (bottom right) represents utilities (*u*_t_ and *u*_r_) for which the improvement in Q-TWiST is statistically significant for Tam alone (*P* ⩽ 0.05). S=significant and NS=not significant.

display similar threshold plots for the ER-negative and ER-positive cohorts, respectively. For patients with ER-negative tumours, CMF followed by Tam is favoured for all values of *u*_t_ and a broad range of *u*_r_. For patients with ER-positive tumours, there was no benefit from the chemotherapy in terms of Q-TWiST. The trend towards an improvement in Tam alone reaches statistical significance in a small area defined by very low values of *u*_t_ and high values of *u*_r_.

Finally, we performed a sensitivity analysis for a range of proposed *α*'s used to transform the SHE scale to reflect a TTO scale. After using an *α* of 1.4, the utilities for each of the three health states were similar although slightly reduced. After using an *α* value of 1.8, again the utilities were similar for all three health states although slightly higher. Thus, incorporating utilities using an *α* within the range of 1.4–1.8 would not markedly affect the results obtained from our Q-TWiST analysis (data not shown).

## DISCUSSION

The main objective of these analyses was to link QL and QAS in comparing the randomised treatments. In addition, we investigated whether there was a difference in the magnitude of the chemotherapy effect according to ER status of the primary tumour as was found for survival in this trial (International Breast Cancer Study Group, 2002).

### Quality of life

The subjective impact of adjuvant therapy was investigated over the first 24 months of treatment. At month 3, patients receiving CMF reported the expected side effects. Better QL in patients with Tam alone was also indicated by the various global measures of well being, coping and health status, despite the earlier beginning of hot flushes for the Tam alone group.

After completing chemotherapy, QL scores rapidly improved as found in our previous trials (Hürny *et al*, 1996b; The International Breast Cancer Study Group, 2001) and in an adjuvant trial by the Eastern Cooperative Oncology Group with a more comprehensive QL questionnaire (Fairclough *et al*, 1999). Contrary to our hypothesis, patients' perception of chemotherapy was not affected by the ER status of their tumour.

We faced problems with the timing of administration for the baseline QL assessment. A substantial proportion of patients completed the QL form after randomisation but before starting CMF. These patients were presumably aware of their assigned treatment. The baseline scores may reflect an anticipation of cytotoxic side effects or perception of worse health status, as described for indicators of QL and health status and for preference measures (Hürny *et al*, 1994; Hürny *et al*, 1996b; Jansen *et al*, 2001b). To eliminate any differential anticipatory effects on baseline scores in future studies, we have introduced a completed QL form as an eligibility criterion.

Overall, the improvement in QL over the first 6 months was more pronounced than the transient impairment by chemotherapy. These findings confirm those of IBCSG Trials VI and VII (Hürny *et al*, 1996b), suggesting that a patient's psychological adaptation is more important for her QL than cytotoxic side effects. There were no residual effects of CMF as assessed by our indicators. Similarly, in a survey in premenopausal women with node-negative breast cancer treated with or without adjuvant CMF, there were no long-term effects of CMF in general and breast cancer-specific QL domains (Joly *et al*, 2000). This finding does not exclude long-term sequelae, such as fatigue (Bower *et al*, 2000), or impaired sexual (Broeckel *et al*, 2002) or cognitive functioning (Phillips and Bernhard, 2003), in subgroups.

### Quality-adjusted survival

The Q-TWiST method has hitherto used utilities assigned arbitrarily (Goldhirsch *et al*, 1989) or estimated based on patient reported QL (Fairclough *et al*, 1999). Although this approach provides a useful decision aid via a threshold analysis, we wanted to take into account patients' own perception of their health status.

We used a global indicator for health status to reflect the patients' perception across the three distinct health states (Tox, TWiST, Rel) (Hürny *et al*, 1998). This extension of the Q-TWiST model has provided new information for decision-making. The extent of impairment during Tox was less severe than the conventional assumptions. This finding is not related to the timing of the baseline assessment as we used only the scores at month 3. Our finding is consistent with those of preference studies showing that a majority of patients who previously received adjuvant chemotherapy for breast cancer accept the morbidity of these therapies in turn for a relatively modest survival gain (Lindley *et al*, 1998; Ravdin *et al*, 1998; Jansen *et al*, 2001a; Simes and Coates, 2001).

The SHE scores during the TWiST health state were less than ‘perfect’ and relatively close to the Tox scores. The fact of having cancer and late effects of surgical and systemic treatments may have had an impact on the health estimates (Broeckel *et al*, 2000; Ganz *et al*, 2002). All patients received Tam. Endocrine side effects may be under-reported (Fellowes *et al*, 2001), especially vasomotor and gynaecological symptoms (Day *et al*, 1999; Fallowfield *et al*, 2001).

The SHE scores during the Rel state were distinctly worse than those of Tox and TWiST, although better than the arbitrary values used in previous analyses. For a majority, the relatively high value may reflect their readjustment and the beneficial impact of the treatment for advanced disease (Bernhard *et al*, 1999). However, there was substantial variability due to both the small number of patients who had a recurrence and the limited information available on these cases.

Overall Q-TWiST accumulated within 72 months after randomisation indicated an advantage of 1 month longer of ‘perfect health’ with CMF rather than Tam alone. However, in the ER-negative cohort, three cycles CMF provided 3 months more of Q-TWiST than Tam alone, while for the larger ER-positive cohort, CMF provided no benefit.

The findings of the QAS analysis thus complement and expand those of the QL investigation. They provide additional information for clinical policy and decision-making in postmenopausal patients with node-negative breast cancer: Taking patients' view into account, patients benefited substantially from adjuvant chemotherapy if their ER status was negative.

These analyses are truncated at 72 months follow-up. Further follow-up will enhance any advantage of chemotherapy over Tam alone.

In contrast to other studies using global indicators of health status for QAS analysis (Earle *et al*, 2000), we specified the frame of individual reference. Patients were asked to imagine they would have to live the rest of their life in their current condition and then to rate their concurrent condition. Although not a true preference measure, this indicator is suitable for large-scale phase-III trials taking into account patients' own evaluation of distinct health states within a given context (e.g. culture). Our premise was to evaluate estimates based on actual experience instead of hypothetical scenarios. Experience of breast cancer (Ashby *et al*, 1994) and adjuvant chemotherapy (Lindley *et al*, 1998; Jansen *et al*, 2001b) have been shown to be associated with current preferences. Most often, this type of evaluation is based on cross-sectional comparisons (Earle *et al*, 2000). A repeated formal utility interview is not feasible in an international phase-III trial and would not imply *a priori* a better performance than a rating scale such as our SHE indicator (Giesler *et al*, 1999).

The method of insertion of utility values into decision models by multiplication of utilities and time assumes the independence of time and values. This assumption has been challenged (Bleichrodt and Johannesson, 1997; Bala *et al*, 1999; Stiggelbout *et al*, 1995; Bernhard *et al*, 2001). Preference studies in cancer patients with repeated assessment reported both reasonably stable (O'Connor *et al*, 1987; Llewellyn-Thomas *et al*, 1993; Jansen *et al*, 2001b) and unstable (Llewellyn-Thomas *et al*, 1992; Jansen *et al*, 2000) utilities, suggesting caution in interpreting single point estimates. Variation in self-report health status across distinct clinical situations is plausible and argues for a longitudinal evaluation.

Further developments include the relationship between additional QL domains, such as fatigue (Sadler and Jacobsen, 2001) and cognitive function (Phillips and Bernhard, 2003), and the SHE-scores across different health states (Lowy and Bernhard, 2004). Finally, this concept can be adapted to the palliative setting where there are no clearly separated health states (Glasziou *et al*, 1998; Cole *et al*, 2004).

In summary, patients receiving initial CMF indicated the expected adverse impact on QL and a delay in adaptation compared to those assigned Tam alone. The patient estimated utilities for TWiST indicated less than ‘perfect health’ under Tam. Longitudinal evaluations of SHE scores provide important information for QAS (Q-TWiST) analysis and clinical decision-making.

## Appendix A. Trial IX

### INTERNATIONAL BREAST CANCER STUDY GROUP

Participants and Authors

*Scientific Committee:* A Goldhirsch, AS Coates (Co-Chairs); *Foundation Council:* J Collins (Pres.), B Thürlimann (VP), H-J Senn (Treasurer), S Holmberg, J Lindtner, A Veronesi, H Cortés-Funes; *Coordinating Center, Bern, Switzerland:* M Castiglione-Gertsch (CEO and Study Chair), ML Nasi (Studies Coordinator), G Egli, M Rabaglio, R Maibach, M Iannino Gerber, A Hiltbrunner; *Statistical Center, Harvard School of Public Health and Dana-Farber Cancer Institute, Boston, MA, USA:* R Gelber (Group Statistician), K Price (Scientific Director), M Bonetti, H Peterson, D Zahrieh, M Zelen, S Gelber, A O’Neill, H Litman; *Data Management Center, Frontier Science & Tech. Res. Found., Amherst, NY, USA:* R Hinkle, M Isley, L Blacher (Director), S Lippert, J Celano; **Pathology Office:** B Gusterson, R Bettelheim, R Reed, G Viale, E Mallon; *Quality of Life Office:* J Bernhard, Ch Hürny, H Gusset, N Mathys, B Cliffe; *Centro di Riferimento Oncologico, Aviano, Italy:* D Crivellari, S Monfardini, E Galligioni, MD Magri, A Veronesi, A Buonadonna. S Massarut, C Rossi, E Candiani, A Carbone, R Volpe, M Roncadin, M Arcicasa, F Coran, S Morassut; *Spedali Civili & Fondazione Beretta, Brescia, Italy:* E Simoncini, G Marini, P Marpicati, M Braga, P Grigolato, L Lucini; *General Hospital, Gorizia, Italy:* S Foladore, L Foghin, G Pamich, C Bianchi, B Marino, A Murgia, V Milan; *European Institute of Oncology, Milano, Italy:* A Goldhirsch, M Colleoni, G Martinelli, L Orlando, F Nolé, A Luini, R Orecchia, G Viale, F Peccatori, F de Braud, A Costa, S Zurrida, P Veronesi, V Sacchini, V Galimberti, M Intra, U Veronesi; *Ospedale Infermi, Rimini, Italy:* A Ravaioli, D Tassinari, G Oliverio, F Barbanti, P Rinaldi, L Gianni, G Drudi; *Ospedale S Eugenio, Roma, Italy:* M Antimi, M Minelli, V Bellini, R Porzio, E Pernazza, G Santeusanio, LG Spagnoli; *Ospedale S. Bortolo, Vicenza, Italy:* M Magazu, V Fosser, P Morandi, G Scalco, M Balli, M Gion, S Meli, G Torsello; *Swiss Group for Clinical Cancer Research (SAKK) member institutions – Inselspital, Bern, Switzerland:* MF Fey, M Castiglione-Gertsch, E Dreher, H Schneider, S Aebi, J Ludin, G Beck, A Haenel, JM Lüthi, HJ Altermatt, M Nandedkar, K Buser; *Kantonsspital, St Gallen, Switzerland:* HJ Senn, B Thürlimann, Ch Oehlschlegel, G Ries, M Töpfer, U Lorenz, O Schiltknecht, B Späti, A Ehrsam, M Bamert, WF Jungi; *Istituto Oncologico della Svizzera Italiana, Bellinzona, Switzerland:* F Cavalli, O Pagani, H Neuenschwander, L Bronz, C Sessa, M Ghielmini, T Rusca, P Rey, J Bernier, E Pedrinis, T Gyr, L Leidi, G Pastorelli, G Caccia, A Goldhirsch; *Kantonsspital, Basel, Switzerland:* R Herrmann, CF Rochlitz, JF Harder, S Bartens, U Eppenberger, J Torhorst; *Hôpital des Cadolles, Neuchâtel, Switzerland:* D Piguet, P Siegenthaler, V Barrelet, RP Baumann; *Kantonsspital, Zürich, Switzerland:* B Pestalozzi, C Sauter, D Fink, M Fehr, U Haller, U Metzger, P Huguenin, R Caduff; *Centre Hospitalier Universitaire Vandois, Lausanne, Switzerland:* L Perey, S Leyvraz, P Anani, F Gomez, D Wellman, G Chapuis, P De Grandi, P Reymond, M Gillet, JF Delaloye; *Hôpital Cantonal, Geneva, Switzerland:* P Alberto, H Bonnefoi, P Schäfer, F Krauer, M Forni, M Aapro, R Egeli, R Megevand, E Jacot-des-Combes, A Schindler, B Borisch, S Diebold; *Kantonsspital Graubünden, Chur, Switzerland:* F Egli, P Forrer, A Willi, R Steiner. J Allemann, T Rüedi, A Leutenegger, U Dalla Torre; *Australian New Zealand Breast Cancer Trials Group (ANZ BCTG) member institutions – Operations Office, University of Newcastle:* JF Forbes, D Lindsay, A Wilson; *Statistical Center, NHMRC CTC, University of Sydney:* RJ Simes, H Dhillon; *The Cancer Council Victoria (previously Anti-Cancer Council of Victoria), Australia:* J Collins, R Snyder, E Abdi, R Basser, WI Burns, M Chipman, J Chirgwin, R Drummond, P Francis, M Green, P Gregory, S Hart, M Henderson, P Kitchen, R McLennan, C Murphy, S Neil, M Pitcher, G Richardson, A Rodger, M Schwarz, P Mitchell, D Joseph, J Griffiths, R Stanley, P Jeal, I Olver, J Zalcberg, G Lindeman, D Finkelde, G Goss, M Steele, B Mann, A Read, I Russell, J Stewart, C Underhill, D Reading; *Auckland Breast Cancer Study Group, Auckland, New Zealand:* RG Kay, VJ Harvey, CS Benjamin, P Thompson, A Bierre, M Miller, B Hochstein, A Lethaby, J Webber, D Porter; *Flinders Medical Centre, Bedford Park, South Australia:* T Malden; Mount Hospital, Perth, Western Australia: G Van Hazel; *Newcastle Mater Misericordiae Hospital Waratah, Newcastle, Australia:* JF Forbes, J Stewart, D Jackson, R Gourlay, J Bishop, S Cox, S Ackland, A Bonaventura, C Hamilton, J Denham, P O’Brien, M Back, S Brae, R Muragasu; *Prince of Wales, Randwick, NSW, Australia:* C Lewis; *Royal Adelaide Hospital, Adelaide, Australia:* IN Olver, D Keefe, M Brown, PG Gill, A Taylor, E Yeoh, E Abdi, J Cleary, F Parnis; *Sir Charles Gairdner Hospital, Nedlands, Western Australia:* M Byrne, G Van Hazel, J Dewar, M Buck, G Sterrett, D Ingram, D Hastrich, D Joseph, F Cameron; *University of Sydney, Dubbo Base Hospital and Royal Prince Alfred Hospital, Sydney, Australia:* MHN Tattersall, AS Coates, F Niesche, R West, S Renwick, J Donovan, P Duval, RJ Simes, A Ng, D Glenn, RA North, J Beith, RG O’Connor, M Rice, G Stevens, J Grassby, S Pendlebury, C McLeod, M Boyer, A Sullivan, J Hobbs, D Lind; *Waikato Hospital, Hamilton, New Zealand:* I Kennedy, G Round, J Long; *West Swedish Breast Cancer Study Group, Göteborg, Sweden:* CM Rudenstam, A Wallgren, S Ottosson-Lönn, R Hultborn, G Colldahl-Jäderström, E Cahlin, J Mattsson, SB Holmberg, L Ivarsson, O Ruusvik, LG Niklasson, S Dahlin, G Karlsson, B Lindberg, A Sundbäck, S Bergegårdh, H Salander, C Andersson, M Heideman, Y Hessman, O Nelzén, G Claes, T Ramhult, JH Svensson, P Liedberg, M Suurküla, S Persson; *Groote Schuur Hospital and University of Cape Town, Cape Town, Rep. of South Africa:* DM Dent, A Gudgeon, E Murray, ID Werner, P Steynor, J Toop, E McEvoy; *Sandton Oncology Center, Johannesburg, Rep. of South Africa:* D Vorobiof, M Chasen, G Fotheringham, G de Muelenaere, B Skudowitz, C Mohammed, A Rosengarten; *The Institute of Oncology, Ljubljana, Slovenia:* J Lindtner, D Erzen, E Majdic, B Stabuc, A Plesnicar, R Golouh, J Lamovec, J Jancar, I Vrhoved, M Kramberger; *Madrid Breast Cancer Group, Madrid, Spain:* H Cortés-Funes, D Mendiola, J Hornedo, R Colomer, F Cruz Vigo, P Miranda, A Sierra, F Martinez-Tello, A Garzon, S Alonso, A Ferrero.
